# 

**Published:** 1912-08-10

**Authors:** 


### Appointments.

Mr. Alexander Macphail, M.B., C.M. (Glasgow), has been appointed lecturer on anatomy to St. Bartholomew's Hospital. He graduated at Glasgow University, and has held a similar post at Charing Cross Hospital.

Mr. S. W. Daw, M.B., B.S., F.R.C.S., of 29 Park Square, Leeds, recently resident surgical officer at the General Infirmary, has been appointed surgical tutor to Leeds University and surgical registrar to the Leeds General Infirmary.

Mr. A. Garrick Wilson, F.R.C.S., has been appointed senior honorary assistant surgeon to the Royal Hospital, Sheffield. Mr. Wilson is a graduate of Cambridge University, and has held appointments at the Sheffield Children's Hospital, the Hospital for Sick Children, Great Ormond Street, London, and at the Halifax Royal Infirmary. His promotion has created a second vacancy on the honorary assistant surgical staff.

### Buildings Contemplated and in Progress.

Aberdeen.—New buildings are projected for the Royal Hospital for Children. Lord and Lady Cowdray have promised £5,000 towards the cost provided a certain sum is subscribed during 1913.

Amersham.—The Rural District Council have resolved to invite plans in competition for an isolation hospital to serve the district.

Bootle.—Bootle Town Council have decided to build an infectious hospital at an estimated cost of £3,600.

Bradford.—Mr. F. Holland, of Bradford, is architect for workhouse buildings to be erected at Daisy Hill for the Guardians. Particulars of tender may be obtained from the architect, and the accepted builder will be required to sign a fair-wages contract clause.

Brentwood.—Alterations and additions are projected to the County Mental Hospital, and the Council have accepted a tender of £2,575 for the work.

Croydon.—Mr. Frank Windsor, architect, prepared the design for and superintended the construction of the Edward VII. Memorial Wing of the Croydon General Hospital, which has just been formally opened.

Dublin.—The Dublin Joint Hospital Board have approved plans for the extension of the present sanatorium accommodation and for a new sanatorium for advanced cases.

Glasgow.—Extensive remodelling is projected to the Glasgow Western Infirmary pathological block. Messrs. John Burnet & Son were architects for the original buildings, and the projected work is estimated to cost £8,000.

Holywell.—At an estimated cost of £8,000 the Guardians have decided to carry out alterations and additions to the workhouse.

Hornsey.—The Borough Engineer, Mr. E. J. Lovegrove, has prepared plans for a new steam laundry block and disinfector at the Infectious Hospital, Highgate. An inquiry has been held regarding the application for sanction to a loan of £4,560.

Lambeth.—St. Thomas's Hospital has benefited by a gift of £20,000 to cover the cost of a new out-patients' department. The donor elects to remain unnamed.

Lower Clapton.—Mr. A. Gordon, of Queen Victoria Street, E.C., is architect for a Mothers' Home which is being erected at Lower Clapton for the Salvation Army Council. The foundation-stone has been formally laid and the work is proceeding rapidly.

### BUREAU OF INFORMATION.

### Rules for Correspondents.

1. Every letter, on whatever subject, must be accompanied by the coupon to be cut from the back cover (inside page) of The Hospital, current issue, and must contain the name and address of the correspondent with pseudonym for publication if desired.
2. Replies by post cannot be given, save under exceptional circumstances at the Editor's discretion.
3. Letters from Approved Homes in reply to special needs published in the Bureau should state terms and full particulars, and be sent prepaid under cover to the Editor of the Bureau with coupon and name enclosed for identification.
4. Proprietors of Homes which have not yet been entered on the List of Approved Homes, but have spare accommodation likely to suit special needs, are invited to write for an application form for registration. The fee for registration is 5s.
5. Special needs of every description relating to those who are sick or in difficulties are made known in these columns free of charge.
6. All communications to be addressed to The Editor of The Hospital, 28 Southampton Street, Strand, London, W.C., and marked "Bureau of Information."

### The Union of Nursing Homes.

All new methods require patience and time to make them work smoothly. The new means we are adopting in these columns to draw the owners of Nursing Homes with spare beds into touch with invalids who are anxious to fill them are hardly yet fully understood. Their success depends on co-operation. When announcements appear in the Bureau from time to time of accommodation needed by persons able to pay moderate terms, it rests with owners of Homes on the Approved List to write and make an offer. But we fear that owners of Homes have got out of the habit of answering announcements, because they frequently find the only response is from some agent who takes a high fee and perhaps has no patient in waiting. What we want the Approved Homes to recognise is that we reserve a section of the Bureau exclusively for them. In this they can make known the advantages they offer to patients. In this free of charge the requirements of patients are made known. It rests with the Homes to make prompt and businesslike response to the members of the public who approach them through the Bureau, and to this end they should study the columns week by week to ascertain whether they contain some case suitable for their Home. Since the Bureau was started we have more than once had occasion to deplore the want of keenness on the part of owners of Homes in responding to the needs made known, resulting in the disappointment of the applicant. We are sure that this results simply because the principle of self-help upon which the Bureau depends has been imperfectly apprehended. We want owners of Homes on the List to realise that it is on them the onus rests of supplying the needs we make known. Patients have discontinued advertising for Nursing Homes owing to the unsatisfactory nature of the replies usually received. It is for the Homes on the Approved List to bring confidence to members of the medical and nursing profession, and of the public who appeal to us for help, by responding instantly to their announcements.

All letters should be sent in a separate stamped envelope enclosed under cover to the Editor, marked "Bureau," and should be accompanied by name of sender for identification and a coupon cut from the current issue. These letters are stamped with the words "Approved Home," and are forwarded without delay.

### Approved Homes.

Note.—*No Home is added to the List without direct medical and other testimony to its good management.*

### For Middle-class Patients.

3 Sudeley Street, Brighton.—Proprietor, Nurse Dunkley, trained maternity nurse. Maternity, medical, and convalescent cases. An excellent Home for patients of moderate means; highly recommended for scrupulous cleanliness and kind attention. Every effort made to suit circumstances of patients.

### Forms of Treatment.

### Electricity Applied to Feet.

Nurse L. J.—The current was certainly not excessive in the case described, and the patient would be well advised not to attempt to obtain compensation. Current, applied by the method you describe, enters the body through the hand holding the positive electrode, passes through the trunk, and leaves through the hand holding the negative electrode. Very little of this current would act directly on the muscles of the feet, but the latter would benefit indirectly as a result of the general toning up of the central nervous system by the electric current. The amount of current and its duration should be ordered by a doctor. Current may be satisfactorily applied directly to the feet by means of unipolar baths. Two earthenware basins containing water are used, and the patient sits on a chair with one foot immersed in each basin. The water in the right basin is connected with the positive electrode, that in the left basin with the negative electrode, therefore the current passes up the right leg, through the pelvis and down the left leg. A current of from 10 to 30 milliamperes (depending upon the nature of the case) may be allowed to flow for ten minutes in this direction; it should then be gradually turned off, the electrodes reversed, and the current switched on again and allowed to flow for ten minutes in the opposite direction.

### Needs and Helpers.

### Fresh-air Home for Delicate Boy.

Anne L.—The child you are interested in needs a long period of good food and airy surroundings, and we may be able to help you to procure it for him if you will send us further particulars. We have in view a small private Home where boys from good homes are received for several months at a time and sent to the village school. The age preferred is between seven and ten. There is no charge.

### Inexpensive School for Deaf Mute.

Zeta.—Write to the Royal School for Deaf and Dumb Children, Margate. Children are received here for thorough training between the ages of seven and ten, and, in addition to a good general education, are taught lip reading and many useful trades. The charge is only £35 a year. The leaving age is sixteen, and apprentice fees are granted to ensure a satisfactory after-career.

### Letter-Box.

V. M.—It is extraordinary that you have never looked for the detailed reply which we gave to your second letter in our issue of July 20. It must be well understood that we do not give replies by post. You will forgive our reminding you that misuse of the Bureau gives needless trouble both to you and to ourselves.

Secretary Sister.—We are greatly obliged for the particulars you send us of your Homes and have filed them for reference. It is sad to hear that you are compelled to refuse on an average three chronic cases a day, and points to the need for a great expansion of chronic relief to sufferers able to pay a small amount.

Woodside House.—We are sorry that you are unable to receive the patient in whom we are interested. Many thanks for particulars.

Sister Pam.—We are very pleased to enter your name as accredited correspondent for your district, and shall be glad to refer to you when occasion arises.

**The leaflet containing particulars of the Bureau is now ready and will be forwarded free on application.**

### Gifts and Bequests.

The Grocers' Company have sent £50 to the National Hospital for Diseases of the Heart, Soho Square.

Under the will of the late Mr. Caleb Bruce Cole the Bristol Royal Infirmary will benefit to the extent of £2,500.

The Chelsea Hospital for Women has received a donation of £100 to its Rebuilding Fund from the Grocers' Company.

The Charing Cross Hospital has received £100 from the Grocers' Company and ten guineas from the Haberdashers' Company.

The Infants Hospital, Vincent Square, Westminster, has received £100 as the result of the *matinée*, given by Lady Holden, of Alston, at the Court Theatre, on the 11th ult.

Mr. John Wainman, of Mapperley Road, Nottingham, has left the ultimate residue of his property to his children in equal shares, whom failing to the Nottingham General Hospital, the Nottingham Children's Hospital, and the Women's Hospital, Castlegate, Nottingham, in equal shares.

Mr. Austen Chamberlain has received £500 from the Chartered Bank of India, Australia, and China for the London School of Tropical Medicine in response to the appeal made by him at the London Chamber of Commerce on Wednesday last week to the banks and other corporations trading in the tropics. The fund, including Lord Wandsworth's bequest, now amounts to nearly £40,000.

### The Opening-up of Bexhill.

The estate of Sir Augustus Webster, Bart., which adjoins Bexhill on the high ground to the west, has hitherto somewhat prevented the development of Bexhill in that direction as it has been used solely for agricultural purposes, and no part of it was therefore available for building. The portion of the property known as the Cooden Wood Estate, which extends to about 120 acres, immediately adjoins the old Sussex village of Little Common, and extends from the main Bexhill to Eastbourne road, which runs along the summit of Cooden Downs in a southerly slope extending almost to the seashore, and in some places immediately adjoining the 18-hole golf links of the Cooden Beach Golf Club, recently formed by Lord De La Warr. As eyes have been cast upon this land for many years by those wishing to build institutions or houses, there should be a great demand for the 147 lots which will be submitted for sale by auction by Messrs. Mabbett and Edge, of Mount Street, Grosvenor Square, London, W., on August 17, at the Kursaal, Bexhill-on-Sea.

### FURNISHING & APPLIANCES OF A COTTAGE HOSPITAL.

An Actual Inventory.

**By the Hon. SYDNEY HOLLAND.**

Second Edition, paper cover. **6d.** net; **7d.** post free.

**Publishers: THE SCIENTIFIC PRESS, LTD., 28 & 29 Southampton Street, Strand, London, W.C.**

*To the Editor of* The Hospital.

*To the Editor of* The Hospital.

### Books Received.

Spottiswoode and Co. Ltd.: "The Dentists Register, 1912;" "The Medical Register, 1912."

Dentists' Manufacturing Co.: "The Prevention of Dental Caries," by Sims Wallace. Second edition.

